# Latin American perceptions of fear and exaggeration transmitted by the media with regard to COVID-19: frequency and association with severe mental pathologies

**DOI:** 10.3389/fpsyg.2023.1037450

**Published:** 2023-05-17

**Authors:** Christian R. Mejia, Telmo Raul Aveiro-Róbalo, Luciana Daniela Garlisi Torales, Verónica Alejandra Alejandra Castro Hidalgo, Jhino Valeriano, David Alfonso Ibarra-Montenegro, Aram Conde-Escobar, Fernanda Sánchez-Soto, Yuliana Canaviri-Murillo, María Oliva-Ponce, Victor Serna-Alarcón, Martín A. Vilela-Estrada, Dennis Arias-Chávez

**Affiliations:** ^1^Translational Medicine Research Centre, Universidad Norbert Wiener, Lima, Peru; ^2^Faculty of Medicine, Universidad del Pacífico, Asunción, Paraguay; ^3^Federación Latinoamericana de Sociedades Científicas de Estudiantes de Medicina (FELSOCEM), Asunción, Paraguay; ^4^Faculty of Medicine, Universidad Hispanoamercana, San José, Costa Rica; ^5^Faculty of Medicine, Universidad Nacional del Altiplano, Puno, Peru; ^6^Faculty of Medicine, Universidad Autónoma de Chiriquí, David, Chiriqui, Panama; ^7^Universidad Privada de Tacna, Centro de Investigación de Estudiantes de Medicina (CIESMED), Tacna, Peru; ^8^Escuela de Medicina Humana, Universidad Privada Antenor Orrego, Trujillo, Peru; ^9^Faculty of Medicine, Universidad Continental, Arequipa, Peru

**Keywords:** media, fear, pandemic, COVID-19, mental health, Latin America

## Abstract

**Introduction:**

The COVID-19 pandemic contributed to the spread of abundant misinformation by the media, which caused fear and concern.

**Objective:**

To determine the association between the pathologies of the mental sphere and the perceptions of fear and exaggeration transmitted by the media with respect to COVID-19 in Latin America.

**Methodology:**

The present study has an analytical cross-sectional design that is based on a validated survey to measure fear and exaggeration transmitted by the media and other sources (Cronbach's α: 0.90). We surveyed more than 6,000 people, originally from 12 Latin American countries, who associated this perceived exaggeration with stress, depression, and anxiety (measured through DASS-21, Cronbach's α: 0.96).

**Results:**

Social networks (40%) or television (34%) were perceived as the sources that exaggerate the magnitude of the events. In addition, television (35%) and social networks (28%) were perceived as the sources that generate much fear. On the contrary, physicians and health personnel are the sources that exaggerated less (10%) or provoked less fear (14%). Through a multivariate model, we found a higher level of global perception that was associated with whether the participant was older (*p* = 0.002), had severe or more serious anxiety (*p* = 0.033), or had stress (*p* = 0,037). However, in comparison with Peru (the most affected country), there was a lower level of perception in Chile (*p* < 0.001), Paraguay (*p* = 0.001), Mexico (*p* < 0.001), Ecuador (*p* = 0.001), and Costa Rica (*p* = 0.042). All of them were adjusted for gender and for those having severe or major depression.

**Conclusion:**

There exists an association between some mental pathologies and the perception that the media does not provide moderate information.

## Introduction

Since the beginning of the pandemic caused by severe acute respiratory syndrome coronavirus 2 (SARS-CoV-2), the media has been playing an important role in the dissemination of information concerning this new virus. Owing to the role played by the media and the rapid spread of the COVID-19 disease in most countries, the disease generated great concern among the populations globally. Even at particular periods of the pandemic, the population worldwide relied greatly on social networks and other means of mass communication to access health information and measures provided by the government to mitigate the spread of the virus (González-Padilla and Tortolero-Blanco, 2020). Although this permits the population to take a more active role in assessing health risks (Román Etxebarrieta et al., 2020), overwhelming and negative information can be detrimental to mental health (Zhong et al., 2021).

In this context, the information disseminated by the media should have been truthful and come from reliable sources. However, in many cases, the information was erroneous and only contributed to generate chaos among the population (Tasnim et al., 2020). In addition to the misinformation, some authority figures generated false statements and/or exaggerated news. For example, the then Brazilian president published a video encouraging self-medication with hydroxychloroquine (Ball and Maxmen, 2020) and in Ayacucho, Peru, the COVID-19 command chief had to be removed from his position after stating, in a press conference, that chlorine dioxide was a recommended drug to treat patients infected with COVID-19 (RPP Grupo, 2021).

In several countries, it was found that the perceptions of COVID-19 fear and/or exaggeration among the population was lower than that in Peru. This could be due to the intrinsic characteristics of the population, as mentioned in a Peruvian study, where certain factors such as being female, older, and religious were associated with fatalistic attitudes, contributing to the belief of erroneous information (Mejia et al., 2020a). Another factor to take into account is that Peru was the country with the worst mortality indicators in the world (Pighi, 2021), which could have had an impact on the way the population perceived the information received.

An investigation carried out by a group of Peruvian nurses found that worry, fear, and having been diagnosed with COVID-19 were predictors of fatalism (Zeladita-Huaman et al., 2022). An interesting study that was carried out in Bolivia reported an association between the levels of knowledge presented by Bolivian medical students according to the fatalistic perception of the disease (Cossio-Andia et al., 2021). Another research conducted among the inhabitants of the USA and the UK revealed that individuals greatly overestimated the peril and transmissibility of COVID-19 compared to expert analysis. However, when provided with proficient information, people's misconceptions regarding the COVID-19 virus were slightly rectified. Furthermore, the study found that the more contagious individuals believed COVID-19 to be, the less likely they were to engage in precautionary measures, which was referred by the researchers as the “fatalism effect” (Akesson et al., 2022).

In a study conducted in China, the results showed that the negative impact of anxiety and fear on doctor–patient trust was mediated using media, which could be explained by a more positive image of doctors during the pandemic. In summary, the study demonstrated the importance of media in the relationship between patients and doctors (Chen et al., 2022). A scientific article investigated the prevalence of conspiracy beliefs surrounding COVID-19 and vaccines among 5,779 individuals across 13 Latin American countries during the pandemic. The findings indicated that, in most countries, women, individuals with lower levels of education, and those who obtained information from family and friends were more likely to endorse conspiratorial ideas concerning the COVID-19 vaccine (Caycho-Rodríguez et al., 2022).

An interesting article concluded that basic personality beliefs, such as the goodness and justice of the world, can act as psychological resources for coping with tough situations in life, such as fear of COVID-19 infection ( Gritsenko et al., 2020). Another research indicated a significant prevalence of mental health issues that were positively linked with frequent social media engagement during the COVID-19 pandemic (Gao et al., 2020). Rodriguez-Besteiro et al. (2023) affirmed in their study that people who had higher levels of anxiety during the confinement by COVID-19 complied more with the confinement, which might have led to a greater consumption of social networks (Rodriguez-Besteiro et al., 2023).

This epidemic of information has been named “infodemic.” It caused fear, speculation, and rumors among the population (Arroyo-Sánchez et al., 2020). In addition, it is presumed that it partly influenced the increase in reported cases of anxiety, stress, and/or depression (Hernández Rodríguez, 2020; Liu and Tong, 2020), as well as distrust in the authorities and health services (Jakovljevic et al., 2020). Therefore, it is necessary to know the population's perceptions with regard to the news and information transmitted by the media during the pandemic, especially in a region as diverse as Latin America. This study aimed to determine the association between mental pathologies and the perceptions of fear and exaggeration transmitted by the media regarding COVID-19 in Latin America.

## Methodology

### Study design and population

An observational, analytical, cross-sectional, and multicenter study was conducted between July 2020 and August 2020. A total of 6,022 inhabitants from 12 Latin American countries were evaluated, including Peru, Chile, Paraguay, Mexico, Colombia, Bolivia, Panama, Ecuador, Costa Rica, El Salvador, Honduras, and Guatemala.

Those who answered the online survey voluntarily, completely, and correctly were included. We excluded the incomplete surveys (those which did not have one or more of the responses about their perception of the media). We discarded 396 surveys for not having answers concerning depression, 167 concerning anxiety, 195 concerning stress, 16 with regard to country, 25 with respect to gender, and 20 for not stating their age. For reasons of logistics and time, it was not possible to replace those discarded surveys.

A non-random, snowball-type sampling was used, in which each respondent requested the participation of family members, friends, fellow students, and acquaintances so that they would share the survey with other people in their environment.

### Instruments and variables

We used the fear perception and magnitude of the issue (MED-COVID-19) scale to measure the perception of fear or exaggeration transmitted by the media (Mejia et al., 2020b), which was self-reported online questionnaire. It consisted of 12 questions with Likert scale-type responses (strongly agree, agree, indifferent, disagree, and strongly disagree). The media scale had a Cronbach's α of 0.90 (with individual α values ranging from 0.89 to 0.90). All the questions had a positive sign and were based on a scale validated in the first months of the pandemic in Peru (Akesson et al., 2022).

In addition, to measure depression, anxiety, and stress, the DASS-21 scale survey was used, which was composed of 21 questions with answers according to a Likert scale-type responses (did not apply to me at all; applied to me to some degree or some of the time; applied to me to a considerable degree or a good part of time; and applied to me very much or most of the time) and other variables were included in the data collection such as gender (male or female), age (in years), and country (within the 12 responses that were taken into account).

### Data collection procedures

Data collection was carried out using Google Forms (this was done when the pandemic was at its worst, between June 2020 and August 2020, when the countries under consideration were facing the first wave and when the information was the most abundant and varied in terms of coverage). After finishing the surveys, a quality control of the data was performed, in which the exclusion criteria were considered. Subsequently, the data collected were coded using Microsoft Excel 2019. Finally, we proceeded with data cleaning and data quality control. Then, the data were exported to the statistical program, Stata (version 11.1).

### Statistical analysis

The analysis was performed using the statistical program Stata (version 11.1). In the first phase, we worked with the categorical variables through descriptive analysis using frequencies/percentages. In the case of numerical variables, medians/interquartile ranges were used. In the second phase, we performed the cross-tabulation of the perception of each one of the items according to the main characteristics to be measured. Generalized linear models (Poisson family, log–link function, and models for robust variances) were used to obtain prevalence ratios, 95% confidence intervals, and *p*-values. A *p*-value of < 0.05 was considered statistically significant.

### Ethical aspects

The project was approved by the research Bioethics Committee of Universidad Privada Antenor Orrego (Ethics Committee Resolution No. 0235-2016-UPAO). In addition, prior to the completion of the virtual questionnaire, their participation in the study was requested through virtual consent. In addition, the information and data collected were treated anonymously, respecting the ethical precepts for scientific research.

## Results

Among the 6,022 respondents in Latin America, significant proportions of them agreed that social networks (39.5%) or television (34.0%) exaggerate the magnitude of the events. In addition, we found that television (35.2%) and social networks (27.5%) generate a lot of fear due to all that had happened. On the contrary, physicians and healthcare personnel are the ones who least exaggerated (10.4%) and provoked fear (14.0%) (Table 1).

**Table 1:** Percentages according to agreeing or strongly agreeing with the perceptions of exaggeration or fear transmitted by the different media with respect to COVID-19 in Latin America.

**Question**	**Agree or completely agree**
The television is exaggerating its magnitude.	2,046 (34.0 %)
Television generates much fear.	2,122 (35.2 %)
Social networks are exaggerating its magnitude.	2377 (39.5 %)
Social networks generate much fear.	1,657 (27.5 %)
Newspapers are exaggerating its magnitude.	1,566 (26.0 %)
Newspapers generate much fear in me.	1,203 (20.0 %)
Radio is exaggerating its magnitude.	1,236 (20.5 %)
Radio generates much fear in me.	963 (16.0 %)
Physicians and health personnel are exaggerating its magnitude.	625 (10.4 %)
Physicians and health personnel generate much fear.	841 (14.0 %)
My family/friends are the ones exaggerating its magnitude.	1,406 (23.4 %)
My family/friends generate much fear in me.	919 (15.3 %)

In the multivariate model, the general perception of fear or exaggeration was associated with a greater overall perception depending on whether the participant was older (*p* = 0.002) and had a more severe or more serious anxiety (*p* = 0.033) or stress (*p* = 0.037). On the contrary, compared to Peru (the most affected country), the perception of fear or exaggeration in Chile (*p* < 0.001), Paraguay (*p* = 0.001), Mexico (*p* < 0.001), Ecuador (*p* = 0.011), and Costa Rica (*p* = 0.042) was lower, all of which were adjusted for sex and having severe or major depression (Table 2).

**Table 2:** Characteristics and factors associated with the general perceptions of fear and exaggeration transmitted by the media during the COVID-19 pandemic in Latin America.

**Variables**	**The most fearful**		**Analytical models**	
	**No** ***n*** **(%)**	**Yes** ***n*** **(%)**	**Bivariate**	**Multivariate**
**Gender**				
Women	2,356 (67.1 %)	1,155 (32.9 %)	Ref.	Ref.
Men	1,625 (64.7 %)	886 (35.3 %)	1.07 (0.99–1.15) 0.053	1.07 (1.00–1.15) 0.058
**Age (years)**	22 (19–27)	22 (19–28)	1.00 (0.99–1.00) 0.348	1.00 (1.00–1.01) 0.002
**Country**				
Peru	2,016 (60.6 %)	1,311 (39.4 %)	Ref.	Ref.
Chile	588 (83.1 %)	120 (16.9 %)	0.43 (0.36–0.51) < 0.001	0.41 (0.35–0.49) < 0.001
Paraguay	339 (69.0 %)	152 (31.0 %)	0.79 (0.68–0.90) 0.001	0.79 (0.69–0.91) 0.001
Mexico	237 (75.0 %)	79 (25.0 %)	0.63 (0.52–0.77) < 0.001	0.64 (0.53–0.78) < 0.001
Colombia	37 (60.7 %)	24 (39.3 %)	1.00 (0.73–1.37) 0.992	0.97 (0.71–1.32) 0.827
Bolivia	213 (66.8 %)	106 (33.2 %)	0.84 (0.72–0.99) 0.038	0.85 (0.73–1.00) 0.052
Panama	86 (67.7 %)	41 (32.3 %)	0.82 (0.63–1.06) 0.126	0.84 (0.65–1.98) 0.165
Ecuador	182 (69.5 %)	80 (30.5 %)	0.77 (0.64–0.93) 0.008	0.78 (0.65–0.94) 0.011
Costa Rica	65 (72.2 %)	25 (27.8 %)	0.70 (0.50–0.99) 0.041	0.70 (0.50–0.99) 0.042
El Salvador	128 (64.3 %)	71 (35.7 %)	0.91 (0.75–1.10) 0.309	0.91 (0.75–1.10) 0.313
Honduras	57 (69.5 %)	25 (30.5 %)	0.77 (0.56–1.08) 0.127	0.80 (0.58–1.11) 0.184
Guatemala	33 (82.5 %)	7 (17.5 %)	0.44 (0.23–0.87) 0.018	0.46 (0.23–0.90) 0.024
**Depression**				
Moderate or <	3,603 (66.8 %)	1,788 (33.2 %)	Ref.	Ref.
Severe or >	378 (59.9 %)	253 (40.1 %)	1.21 (1.09–1.34) < 0.001	1.07 (0.93–1.24) 0.312
**Anxiety**				
Moderate or <	3,488 (67.2 %)	1,700 (32.8 %)	Ref.	Ref.
Severe or >	493 (59.1 %)	341 (40.9 %)	1.25 (1.14–1.37) < 0.001	1.15 (1.01–1.31) 0.033
**Stress**				
Moderate or <	3,624 (66.8 %)	1,799 (33.2 %)	Ref.	Ref.
Severe or >	357 (59.6 %)	242 (40.4 %)	1.22 (1.10–1.35) < 0.001	1.17 (1.01–1.36) 0.037

The descriptive values for age are in the form of the median (interquartile range). Prevalence ratios (95% confidence intervals) and *p*-values were obtained with generalized linear models (Poisson family, log–link function, and models for robust variances). The upper tercile of the score sum of perception of fear and exaggeration transmitted by the pandemic news was taken as the category of interest of the dependent variable.

In the multivariate model, the perception of exaggeration transmitted by the media among men was higher (*p* = 0.001). Conversely, compared to Peru (the most affected country of all), the perception in Chile (*p* < 0.001), Mexico (*p* = 0.007), and Ecuador (*p* = 0.022) was lower, all of which were adjusted for age and suffering from depression, anxiety, or severe or major stress (Table 3).

**Table 3:** Characteristics and factors associated with the perception of exaggeration transmitted by the media during the COVID-19 pandemic in Latin America.

**Variables**	**The most exaggeration perceived**		**Analytical models**	
	**No** ***n*** **(%)**	**Yes** ***n*** **(%)**	**Bivariate**	**Multivariate**
**Gender**				
Women	2,248 (64.0 %)	1,263 (36.0 %)	Ref.	Ref.
Men	1,511 (60.2 %)	1,000 (39.8 %)	1.11 (1.04–1.18) 0.002	1.11 (1.04–1.19) 0.001
**Age (years)**	22 (19–28)	22 (19–27)	1.00 (0.99–1.00) 0.415	1.00 (0.99–1.00) 0.395
**Country**				
Peru	1,953 (58.7 %)	1,374 (41.3 %)	Ref.	Ref.
Chile	551 (77.8 %)	157 (22.2 %)	0.54 (0.47–0.62) < 0.001	0.52 (0.45–0.61) < 0.001
Paraguay	298 (60.7 %)	193 (39.3 %)	0.95 (0.85–1.07) 0.408	0.96 (0.86–1.08) 0.535
Mexico	213 (67.4 %)	103 (32.6 %)	0.79 (0.67–0.93) 0.005	0.80 (0.68–0.94) 0.007
Colombia	32 (52.5 %)	29 (47.5 %)	1.15 (0.88–1.50) 0.301	1.13 (0.86–1.47) 0.379
Bolivia	199 (62.4 %)	120 (37.6 %)	0.91 (0.79–1.05) 0.213	0.92 (0.79–1.07) 0.276
Panama	72 (56.7 %)	55 (43.3 %)	1.05 (0.86–1.28) 0.647	1.06 (0.87–1.30) 0.568
Ecuador	174 (66.4 %)	88 (33.6 %)	0.81 (0.68–0.97) 0.021	0.82 (0.68–0.97) 0.022
Costa Rica	61 (67.8 %)	29 (32.2 %)	0.78 (0.58–1.06) 0.108	0.79 (0.58–1.07) 0.121
El Salvador	125 (62.8 %)	74 (37.2 %)	0.90 (0.74–1.08) 0.267	0.90 (0.75–1.08) 0.272
Honduras	54 (65.9 %)	28 (34.1 %)	0.83 (0.61–1.12) 0.219	0.84 (0.62–1.14) 0.275
Guatemala	27 (67.5 %)	13 (32.5 %)	0.79 (0.50–1.23) 0.295	0.81 (0.52–1.27) 0.351
**Depression**				
Moderate or <	3,395 (63.0 %)	1,996 (37.0 %)	Ref.	Ref.
Severe or >	364 (57.7 %)	267 (42.3 %)	1.14 (1.04–1.26) 0.007	1.06 (0.93–1.21) 0.404
**Anxiety**				
Moderate or <	3,280 (63.2 %)	1,908 (36.8 %)	Ref.	Ref.
Severe or >	479 (57.4 %)	355 (42.6 %)	1.16 (1.06–1.26) 0.001	1.09 (0.97–1.24) 0.152
**Stress**				
Moderate or <	3,413 (62.9 %)	2,010 (37.1 %)	Ref.	Ref.
Severe or >	346 (57.8 %)	253 (42.2 %)	1.14 (1.03–1.26) 0.010	1.00 (0.97–1.00) 0.134

The descriptive values for age are in the form of the median (interquartile range). Prevalence ratios (95% confidence intervals) and *p*-values were obtained with generalized linear models (Poisson family, log–link function, and models for robust variances). We took the upper tercile of the sum of the score of the perceived exaggeration of the pandemic news as the category of interest of the dependent variable.

In the multivariate model, the perception of fear transmitted by the media was more significantly associated with the perception of being older (*p* = 0.004) and having severe or more serious anxiety (*p* = 0.001) or stress (*p* = 0.009). Conversely, men have a lower perception of fear (*p* < 0.001), and compared to Peru (the most affected country), the perception among men in Chile (*p* = 0.001), Paraguay (*p* = 0.005), and Mexico (*p* = 0.029) was lower, all of which were adjusted for having severe or major depression (Table 4).

**Table 4:** Characteristics and factors associated with the perception of fear transmitted by the media during the COVID-19 pandemic in Latin America.

**Variables**	**Most perceived fear**		**Analytical models**	
	**No** ***n*** **(%)**	**Yes** ***n*** **(%)**	**Bivariate**	**Multivariate**
**Gender**				
Women	1,640 (46.7 %)	1,871 (53.3 %)	Ref.	Ref.
Men	1,324 (52.7 %)	1,187 (47.3 %)	0.89 (0.84–0.93) < 0.001	0.90 (0.85–0.94) < 0.001
**Age (years)**	22 (19–27)	22 (19–28)	1.00 (0.99–1.00) 0.157	1.00 (1.00–1.01) 0.004
**Country**				
Peru	1,577 (47.4 %)	1,750 (52.6 %)	Ref.	Ref.
Chile	370 (52.3 %)	338 (47.7 %)	0.91 (0.83–0.99) 0.023	0.87 (0.80–0.94) 0.001
Paraguay	265 (54.0 %)	226 (46.0 %)	0.88 (0.79–0.97) 0.010	0.86 (0.78–0.96) 0.005
Mexico	169 (53.5 %)	147 (46.5 %)	0.88 (0.78–0.99) 0.049	0.87 (0.77–0.99) 0.029
Colombia	27 (44.3 %)	34 (55.7 %)	1.06 (0.85–1.33) 0.615	1.02 (0.82–1.27) 0.874
Bolivia	156 (48.9 %)	163 (51.1 %)	0.97 (0.87–1.09) 0.612	0.96 (0.86–1.07) 0.483
Panama	68 (53.5 %)	59 (46.5 %)	0.88 (0.73–1.08) 0.199	0.90 (0.74–1.08) 0.259
Ecuador	134 (51.2 %)	128 (48.8 %)	0.93 (0.82–1.06) 0.258	0.93 (0.82–1.06) 0.271
Costa Rica	44 (48.9 %)	46 (51.1 %)	0.97 (0.79–1.19) 0.783	0.97 (0.79–1.19) 0.764
El Salvador	91 (45.7 %)	108 (54.3 %)	1.03 (0.90–1.18) 0.641	1.04 (0.92–1.19) 0.510
Honduras	40 (48.8 %)	42 (51.2 %)	0.97 (0.79–1.21) 0.807	0.99 (0.80–1.23) 0.959
Guatemala	23 (57.5 %)	17 (42.5 %)	0.81 (0.56–1.16) 0.248	0.82 (0.57–1.19) 0.305
**Depression**				
Moderate or <	2,729 (50.6 %)	2,662 (49.4 %)	Ref.	Ref.
Severe or >	235 (37.2 %)	396 (62.8 %)	1.27 (1.19–1.36) < 0.001	1.08 (0.98–1.18) 0.112
**Anxiety**				
Moderate or <	2,656 (51.2 %)	2,532 (48.8 %)	Ref.	Ref.
Severe or >	308 (36.9 %)	526 (63.1 %)	1.29 (1.22–1.37) < 0.001	1.16 (1.06–1.26) 0.001
**Stress**				
Moderate or <	2,753 (50.8 %)	2,670 (49.2 %)	Ref.	Ref.
Severe or >	211 (35.2 %)	388 (64.8 %)	1.32 (1.23–1.40) < 0.001	1.14 (1.03–1.26) 0.009

The descriptive values for age are in the form of the median (interquartile range). Prevalence ratios (95% confidence intervals) and *p*-values were obtained with generalized linear models (Poisson family, log–link function, and models for robust variances). We considered the upper tercile of the sum of the score of perceived fear of the pandemic news as the category of interest of the dependent variable.

In the multivariate model, the perceptions of fear and exaggeration transmitted by healthcare professionals or family/friends among men (*p* < 0.001) and those having severe or more severe depression (*p* = 0.001) was higher. Conversely, compared to Peru (the most affected country), there were lower perceptions of fear and exaggeration transmitted by healthcare professionals or family/friends in Chile (*p* < 0.001), Paraguay (*p* < 0.001), Mexico (*p* < 0.001), Colombia (*p* = 0.041), Bolivia (*p* = 0.003), Panama (*p* = 0.005), Ecuador (*p* = 0.021), El Salvador (*p* = 0.001), Honduras (*p* = 0.016), and Guatemala (*p* = 0.034), all of which were adjusted for age and having severe or major anxiety or stress (Table 5).

**Table 5:** Characteristics and factors associated with fear and exaggeration transmitted by healthcare professional or family/friends during the COVID-19 pandemic in Latin America.

**Variables**	**More fear or exaggeration**		**Analytical models**	
	**No** ***n*** **(%)**	**Yes** ***n*** **(%)**	**Bivariate**	**Multivariate**
**Gender**				
Women	2,129 (60.6 %)	1,382 (39.4 %)	Ref.	Ref.
Men	1,354 (53.9 %)	1,157 (46.1 %)	1.18 (1.10–1.24) < 0.001	1.17 (1.10–1.24) < 0.001
**Age (years)**	22 (19–28)	21 (19–26)	0.99 (0.99–0.99) < 0.001	0.99 (0.99–1.00) 0.105
**Country**				
Peru	1,691 (50.8 %)	1,636 (49.2 %)	Ref.	Ref.
Chile	553 (78.1 %)	155 (21.9 %)	0.45 (0.39–0.51) < 0.001	0.44 (0.38–0.51) < 0.001
Paraguay	305 (62.1 %)	186 (37.9 %)	0.77 (0.68–0.87) < 0.001	0.78 (0.70–0.88) < 0.001
Mexico	197 (62.3 %)	119 (37.7 %)	0.77 (0.66–0.89) < 0.001	0.77 (0.67–0.89) < 0.001
Colombia	39 (63.9 %)	22 (36.1 %)	0.73 (0.52–1.02) 0.070	0.71 (0.51–0.99) 0.041
Bolivia	194 (60.8 %)	125 (39.2 %)	0.80 (0.69–0.92) 0.002	0.81 (0.70–0.93) 0.003
Panama	83 (65.4 %)	44 (34.6 %)	0.70 (0.55–0.90) 0.004	0.71 (0.56–0.90) 0.005
Ecuador	152 (58.0 %)	110 (42.0 %)	0.85 (0.74–0.99) 0.034	0.84 (0.73–0.97) 0.021
Costa Rica	56 (62.2 %)	34 (37.8 %)	0.77 (0.59–1.00) 0.053	0.79 (0.60–1.03) 0.079
El Salvador	129 (64.8 %)	70 (35.2 %)	0.72 (0.59–0.87) 0.001	0.72 (0.60–0.87) 0.001
Honduras	55 (67.1 %)	27 (32.9 %)	0.67 (0.49–0.91) 0.011	0.68 (0.50–0.93) 0.016
Guatemala	29 (72.5 %)	11 (27.5 %)	0.56 (0.34–0.93) 0.024	0.58 (0.35–0.96) 0.034
**Depression**				
Moderate or <	3,176 (58.9 %)	2,215 (41.1 %)	Ref.	Ref.
Severe or >	307 (48.7 %)	324 (51.3 %)	1.25 (1.15–1.36) < 0.001	1.21 (1.08–1.35) 0.001
**Anxiety**				
Moderate or <	3,059 (59.0 %)	2,129 (41.0 %)	Ref.	Ref.
Severe or >	424 (50.8 %)	410 (49.2 %)	1.20 (1.11–1.29) < 0.001	1.06 (0.96–1.18) 0.261
**Stress**				
Moderate or <	3,170 (58.5 %)	2,253 (41.5 %)	Ref.	Ref.
Severe or >	313 (52.3 %)	286 (47.7 %)	1.15 (1.05–1.26) 0.002	1.07 (0.94–1.20) 0.316

The descriptive values for age are in the form of the median (interquartile range). Prevalence ratios (95% confidence intervals) and p-values were obtained with generalized linear models (Poisson family, log–link function, and models for robust variances). We considered the upper tercile of the sum of the score of perceived fear and exaggeration transmitted by healthcare personnel and family/friends in the pandemic as the category of interest of the dependent variable.

## Discussion

The results obtained in this study showed that television and social networks are the media that generate the greatest perception of fear and exaggeration with regard to COVID-19. This was also described by Ramazan and Murad (Ramazan Ahmad and Murad, 2020) who reported that news and information posted on social networks caused panic among the population during the COVID-19 pandemic. In Brazil, a study found that the most used means of information during the COVID-19 pandemic was the Internet and that certain news would have had a negative psychological impact on the population. The months in which there were greater mental health repercussions were the ones when the information disseminated by the media was not controlled (Garcia Filho et al., 2020). This was predictable since untruthful information could influence the mental health of the population. Several studies evaluated the patterns of search and access to pages, showing that most of the population acquired information from the unofficial pages of unconfirmed information (Cuan-Baltazar et al., 2020; Rovetta and Bhagavathula, 2020), which could provide false or unreliable news that could be misleading and, as a whole, caused infodemic among the population.

When the COVID-19 pandemic started, the mental health of the population was fairly affected as there was a constant increase in cases of psychological and mental health problems, which was reported among the population worldwide (Hernández Rodríguez, 2020; Huarcaya-Victoria, 2020). Our study showed that people suffering from anxiety or severe stress had a greater perception of fear or exaggeration of the information transmitted by the media. This may be because people with a high level of anxiety or stress are more susceptible to believing that they could suffer from COVID-19, as they believe that they are concerned with getting this disease and generate an environment of extreme care for not getting infected with the virus. This moderately increased the levels of anxiety or stress in the population, influencing their behavior and their ability to make rational decisions (Huarcaya-Victoria, 2020). There has also been a report of evidence of an increase in the levels of anxiety or stress among the population in the situation related to other scenarios, such as the more cases of anxiety and stress observed during the outbreak of measles in California, which occurred more frequently during the initial stage of the crisis (Meadows et al., 2019). Similarly, a study conducted during the global Zika virus emergency showed that the new disease caused confusion and anxiety in the population due to the risks it posed (Park et al., 2019).

On the contrary, an increase in cases of depression and stress in the population has been reported not only due to the pandemic but also due to some characteristics of the pandemic, such as unemployment, death of family members, and social isolation (Shader, 2020). We found that those who suffered from severe depression had a greater perception of fear and exaggeration transmitted by healthcare professionals or family/friends. It should also be mentioned that, although healthcare personnel provide a variety of information, which is generally considered by the population, there have been cases in which some professionals have recommended treatments that had no scientific support, such as chlorine dioxide (RPP Grupo, 2021) or the administration of ivermectin to treat COVID-19 (Lescano and Pinto, 2020), which have put the health of the population at risk. Thus, people with depression, who have distorted information processing (Dehn and Beblo, 2019), could have a greater perception of fear and exaggeration of the information provided by professionals.

In this study, it was also found that men had a greater perception of exaggeration transmitted by health personnel and family/friends with regard to COVID-19 news. In a Peruvian study with similar results, it had been identified that the female population considered that the information provided by media, health personnel, family, and friends are sources of truthful information. Hence, they did not perceive exaggeration in the messages transmitted by health personnel and family/friends with regard to COVID-19 news (Mejia et al., 2020c). This could mean that men believe that the information is false or manipulated and that is the reason why they describe it as exaggerated. It is important to consider that other factors may be influencing these differences and that the results of this study cannot be generalized to other populations or contexts.

The main limitation of this study was selection bias; due to the non-randomized sampling used in the study, the results could not be extrapolated to the total population of the countries where the surveys were conducted. However, this limitation was partial since the objective of this study was never to extrapolate the results but to search for associations between pathologies of the mental sphere and the perception of fear and exaggeration transmitted by the media during the COVID-19 pandemic. Similarly, despite this limitation, we have included a large sample of respondents from 12 Latin American countries that were among the most affected countries in the world. Therefore, these results should be taken as the baseline, and we should be cautious of what the media transmit in future pandemic events (without interfering with the right of dissemination but knowing that, if this right is misused, it can generate serious mental health problems in the recipients due to fear and exaggeration transmitted by the media).

## Conclusion

It can be inferred from this study that anxiety and severe stress are linked to a heightened perception of fear propagated by the media and that individuals experiencing stress, anxiety, or depression due to the COVID-19 pandemic are more likely to perceive exaggeration in messages propagated by media. Moreover, those suffering from severe depression tend to perceive greater levels of fear and exaggeration not only from healthcare professionals but also from loved ones. It is worth noting that, while healthcare providers disseminate a range of information that is typically heeded by the public, individuals with high levels of anxiety or stress may be more inclined to believe that they are at risk of contracting COVID-19 and therefore adopt extreme precautionary measures to avoid getting infected with the virus.

## Data availability statement

The raw data supporting the conclusions of this article will be made available by the authors, without undue reservation.

## Ethics statement

The studies involving human participants were reviewed and approved by the Research Bioethics Committee of Universidad Privada Antenor Orrego (Ethics Committee Resolution No. 0235-2016-UPAO). The patients/participants provided their written informed consent to participate in this study.

## Author contributions

CM, TA-R, and LG contributed to conception and design of the study. LG and TA-R organized the database and wrote the first draft of the manuscript. CM performed the statistical analysis. VC, JV, DI-M, AC-E, FS-S, YC-M, MO-P, VS-A, MV-E, and DA-C wrote sections of the manuscript. All authors contributed to manuscript revision, read, and approved the submitted version.
